# Clock-dependent chromatin topology modulates circadian transcription and behavior

**DOI:** 10.1101/gad.312397.118

**Published:** 2018-03-01

**Authors:** Jérôme Mermet, Jake Yeung, Clémence Hurni, Daniel Mauvoisin, Kyle Gustafson, Céline Jouffe, Damien Nicolas, Yann Emmenegger, Cédric Gobet, Paul Franken, Frédéric Gachon, Félix Naef

**Affiliations:** 1 School of Life Sciences, Ecole Polytechnique Fédérale de Lausanne (EPFL), CH-1015 Lausanne, Switzerland;; 2 Nestle Institute of Health Sciences, CH-1015 Lausanne, Switzerland;; 3 Center for Integrative Genomics, University of Lausanne, CH-1015 Lausanne, Switzerland

**Keywords:** circadian rhythms, chromatin topology, promoter–enhancer loops, DNA regulatory elements, transcriptional bursting

## Abstract

The circadian clock in animals orchestrates widespread oscillatory gene expression programs, which underlie 24-h rhythms in behavior and physiology. Several studies have shown the possible roles of transcription factors and chromatin marks in controlling cyclic gene expression. However, how daily active enhancers modulate rhythmic gene transcription in mammalian tissues is not known. Using circular chromosome conformation capture (4C) combined with sequencing (4C-seq), we discovered oscillatory promoter–enhancer interactions along the 24-h cycle in the mouse liver and kidney. Rhythms in chromatin interactions were abolished in arrhythmic *Bmal1* knockout mice. Deleting a contacted intronic enhancer element in the *Cryptochrome 1* (*Cry1*) gene was sufficient to compromise the rhythmic chromatin contacts in tissues. Moreover, the deletion reduced the daily dynamics of *Cry1* transcriptional burst frequency and, remarkably, shortened the circadian period of locomotor activity rhythms. Our results establish oscillating and clock-controlled promoter–enhancer looping as a regulatory layer underlying circadian transcription and behavior.

## Abstract (precis)

Using circular chromosome conformation capture (4C-seq), Mermet et al. discovered oscillatory promoter–enhancer interactions along the 24-h cycle in mouse livers and kidneys. Deleting a contacted intronic enhancer element in the *Cryptochrome 1* (*Cry1*) gene reduced the daily dynamics of *Cry1* transcriptional burst frequency and shortened the circadian period of locomotor activity rhythms.

The circadian clock, encoded in a core genetic network, governs rhythms in behavior and physiology (Schibler et al. 2015), such as nocturnal activity in mice and oscillations in carbohydrate and lipid metabolism in the liver (Bass and Lazar 2016). This clock also orchestrates the daily rhythmic synthesis of thousands of transcripts by impinging on multiple gene regulatory layers (Zhang et al. 2014). These rhythmic transcripts often coincide with rhythms in chromatin modifications, DNA accessibility, enhancer activity, and transcription factor (TF) binding at promoter-proximal and promoter-distal regions (Mermet et al. 2017; Takahashi 2017), suggesting that chromatin interactions play a role in regulating circadian gene expression.

Chromatin architecture in the nucleus is organized over multiple scales (Dekker et al. 2013). At the fine scale, this organization involves the interactions between gene promoters and enhancer DNA elements through promoter–enhancer looping (Fulco et al. 2016). The remodeling of such DNA contacts and the accompanying dynamics of transcriptional responses have been investigated in the context of signal-dependent gene induction, cell differentiation, and developmental transitions (Palstra et al. 2003; Ghavi-Helm et al. 2014; Kuznetsova et al. 2015). However, little is known about the dynamics of DNA looping along the recurring daily 24-h cycle and the consequences on clock-dependent gene expression in animals.

Cell culture models investigating genes of interest have suggested that nuclear compartmentalization modulates cyclic gene expression (Zhao et al. 2015) and that oscillatory contacts between gene promoters and genomic regions on *trans* chromosomes accompany rhythmic mRNA expression (Aguilar-Arnal et al. 2013). Recently, we described tissue-specific chromatin interactions selectively associated with rhythmically expressed clock output transcripts (Yeung et al. 2018), but, in general, the circadian dynamics of DNA interactions, including their regulation of core clock function and control of circadian gene expression, remain an open question. Indeed, rhythmic transcription could be regulated over an established static promoter–enhancer network (Ghavi-Helm et al. 2014; Xu et al. 2016), or, conversely, the clock could drive dynamic promoter–enhancer looping for high-amplitude daily oscillations in transcription.

Here we monitored promoter–enhancer contacts of a core clock and metabolic clock output gene across time and genotypes in mouse tissues and discovered that contact frequencies oscillated along the 24-h cycle. In arrhythmic *Bmal1* knockout animals, these oscillations were abolished. Deletion in mice of an enhancer that was rhythmically recruited to the *Cryptochrome 1* (*Cry1*) promoter led to a short period phenotype in locomotor activity. Moreover, this deletion compromised rhythmic chromatin topology in the liver and led to reduced peak *Cry1* mRNA expression levels. Finally, single-molecule RNA fluorescent in situ hybridization (smRNA-FISH) showed that the abolished rhythmic chromatin contact reduced the daily dynamics of *Cry1* transcriptional burst frequency.

## Results

### Rhythmic local chromatin interactions in mouse livers

**Figure 1.**
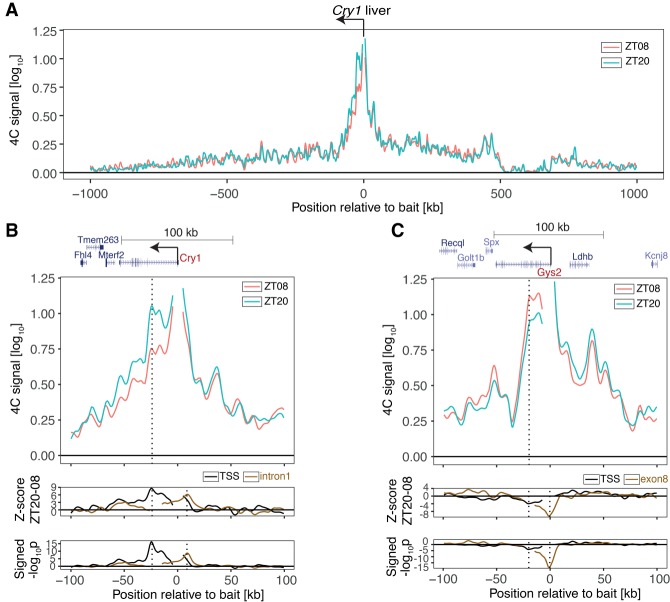
Rhythmic chromatin interactions in mouse livers. (*A*) 4C-seq data (LWMR summarizes *n* = 4 animals per group) in a 2-Mb genomic region surrounding *Cry1* at ZT08 and ZT20. (*B*) 4C-seq signals in a 200-kb genomic region surrounding *Cry1* at ZT08 and ZT20. (*Bottom* tracks) *Z*-score and signed −log_10_(*p*) show rhythmic contacts between the promoter region and the intronic region. (Black) *Cry1* TSS bait (*P* < 10^−16^ at peak); (brown) *Cry1* intron1 bait (*P* < 10^−8^ at peak). (*C*) Same as *B*, targeting the *Gys2* promoter. (*Bottom* tracks) Same as *B* for *Gys2* TSS bait (*P* < 10^−4^ at peak). (Brown) *Gys2* exon8 bait (*P* < 10^−18^ at peak). Vertical dotted lines show the positions locally of maximal differential chromatin interactions.

We focused on two genes representing key temporally regulated hepatic functions: a gene essential for the core circadian oscillator, *Cry1* (Griffin et al. 1999; van der Horst et al. 1999), and a liver-specific clock-controlled gene, *Glycogen Synthase 2* (*Gys2*) (Doi et al. 2010), which encodes the rate-limiting enzyme in hepatic glycogen synthesis (Irimia et al. 2010). These transcripts are rhythmically expressed in the liver at opposite times of day, *Cry1* peaking during the night at Zeitgeber time 20 (ZT20) and *Gys2* peaking during the day at ZT08 (with ZT0 corresponding to lights on and ZT12 corresponding to lights off) (Supplemental Fig. S1A). Using circular chromosome conformation capture (4C) combined with sequencing (4C-seq) (Gheldof et al. 2012), we estimated the interaction frequencies of DNA bait fragments placed near the transcription start sites (TSSs) of *Cry1* and *Gys2* versus the entire genome in livers of wild-type mice collected at ZT08 and ZT20 (*n* = 4 per time point). 4C-seq signals around the *Cry1* and *Gys2* TSSs decayed to background levels following a power law (Supplemental Fig. S1B,C; Supplemental Table S1; Sanborn et al. 2015) and did not exceed background on *trans* chromosomes (Supplemental Fig. S1D,E; Supplemental Table S1). The high proportion of chromatin interactions within the first 2 Mb surrounding the baits on the *cis* chromosome (*Cry1* TSS: 41% of total *cis* contacts at ZT08 and 46% at ZT20; *Gys2* TSS: 54% at ZT08 and 57% at ZT20) indicated that *Cry1* and *Gys2* regulatory contacts were contained within this signal-rich region (Sanyal et al. 2012). To compare 4C-seq profiles across conditions, we normalized the data and applied locally weighted multilinear regression (LWMR), which uses a Gaussian window (σ = 2500 kb) centered on each fragment for local smoothing (Materials and Methods). For *Cry1,* the 4C-seq profiles after LWMR were similar between ZT08 and ZT20 except in a region downstream from the *Cry1* promoter, where the contact frequency was increased at ZT20 (Fig. 1A). While the differential signal covered the entire *Cry1* locus*,* the largest difference was localized—peaking 26 kb downstream from the TSS in the first *Cry1* intron—and highly significant (*P* < 5.5 × 10^−17^ at the peak) (Fig. 1B, bottom tracks, vertical dotted line at the left). A secondary peak was observed near the 3′ end of the *Cry1* transcript.

To further validate the time-dependent contacts, we placed a bait at the +26-kb intronic site (reciprocal 4C-seq). The reciprocal 4C-seq confirmed the increased contact frequency with the *Cry1* promoter region at ZT20 compared with ZT08 (Fig. 1B bottom tracks, brown solid line; Supplemental Fig. S2A). In fact, the reciprocal differential signal peaked 7 kb upstream of the *Cry1* TSS, a site that was also differentially contacted by the *Cry1* TSS bait (*P* < 1.9 × 10^−9^) (Fig. 1B, bottom tracks, vertical dotted line at the right; Supplemental Fig. S2A; Supplemental Table S1). Thus, these 4C-seq data in the liver suggested dynamic contacts between the *Cry1* promoter and the +26-kb intronic site as well as the −7-kb upstream site. Since *Cry1* mRNA accumulated rhythmically in the kidney (Supplemental Fig. S3A), we also performed 4C-seq in kidneys. Consistent with the liver data, these sites were also recruited to the *Cry1* promoter more frequently at ZT20 than at ZT08 (Supplemental Fig. S3B,C).

Opposite to *Cry*1, the *Gys2* promoter contacted an intragenic region more frequently at ZT08 versus ZT20 (Fig. 1C), with a peak 21 kb downstream from the TSS in exon 8 (*P* < 8.7 × 10^−5^ at peak) (Fig. 1C, bottom tracks, black solid line, vertical dotted line at the left), consistent with its anti-phasic rhythmic mRNA accumulation (Supplemental Fig. S1A). This significant differential signal was validated by reciprocal 4C-seq using the exon 8 as bait (*P* < 2.3 × 10^−19^ at peak) (Fig. 1C, bottom tracks, brown solid line, vertical dotted line at the right; Supplemental Fig. S2B). In the kidney, where *Gys2* mRNA accumulation was constant and low, this differential signal was absent (Supplemental Fig. S3D–F). Thus, both gene promoters formed DNA loops with neighboring intragenic regions in *cis* that coincided with the timing of the respective peaks in *Cry1* and *Gys2* mRNA expression.

### The dynamics of chromatin topology depend on BMAL1

**Figure 2.**
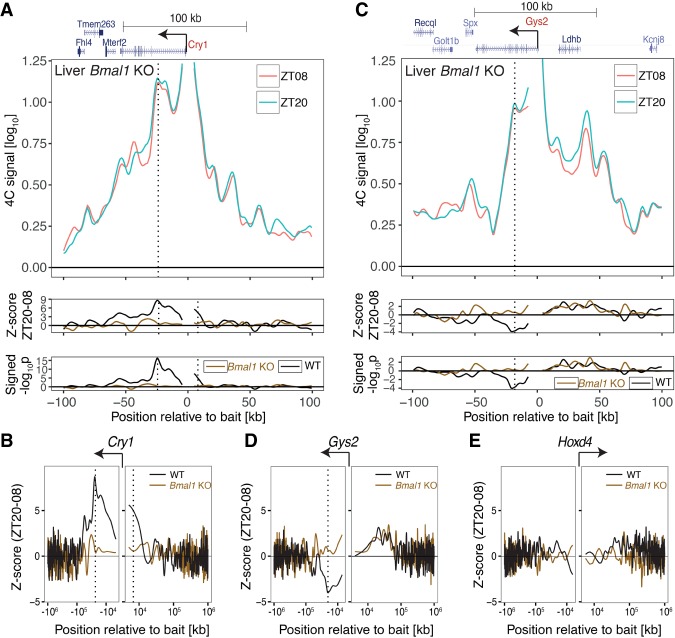
The dynamics of chromatin topology depend on BMAL1. (*A*, *top*) 4C-seq signal targeting *Cry1* from the livers of *Bmal1* knockout mice at ZT20 versus ZT08 shows loss of rhythms in chromatin interactions. (*Bottom*) *Z*-score and signed −log_10_(*p*) of differential 4C-seq signal (ZT20–ZT08) in wild-type versus *Bmal1* knockout. Vertical lines show BMAL1-dependent rhythmic contacts. (*B*) *Z*-score in a 2-Mb genomic region surrounding *Cry1* in wild-type versus *Bmal1* knockout. (*C*) Same as in *A* but for *Gys2* bait. (*D*,*E*) Same as in *B* but for *Gys2* (*D*) and *Hoxd4* (*E*) baits*. B* and *D* show that the BMAL1-dependent rhythmic contacts are localized within 100 kb of the bait.

To test whether these dynamic contacts depended on a functional circadian clock, we performed 4C-seq in the livers of clock-deficient animals (*Bmal1* knockout) in which *Cry1* and *Gys2* lost rhythmic expression and were constantly expressed at high and low levels, respectively (Supplemental Fig. S4A,B). In *Bmal1* knockout, the *Cry1* +26-kb intronic and −7-kb upstream regions contacted the promoter at comparable frequencies at ZT20 and ZT08, suggesting static chromatin loops (Fig. 2A,B). For *Gys2*, the profile between the exon 8 region and the promoter was also static (Fig. 2C,D). Comparing wild-type and *Bmal1* knockout at both time points revealed that for *Cry1*, the loop was locked in a closed conformation (Supplemental Fig. S4C, constitutively high frequencies), and for *Gys2*, it was locked in an open conformation (Supplemental Fig. S4D, constitutively low frequencies). Thus, the closed and open states of DNA loops concurred with high and low transcription, respectively (Supplemental Fig. S4, cf, A,C and B,D). We note that these 4C profiles suggested a BMAL1-independent interaction upstream of *Gys2* (Fig. 2C, lower panels), but this effect was less robust compared with the BMAL1-dependent intragenic looping. As a negative control, we targeted the *Hoxd4* locus, which is a transcriptionally silent region in the adult liver. As expected, chromatin contact profiles at the *Hoxd4* locus remained static over time in both wild-type and *Bmal1* knockout livers (Fig. 2E; Supplemental Fig. S4E). These data thus showed that rhythmic loops in *Cry1* and *Gys2* depended on the clock TF BMAL1.

### Rhythmic DNA loops connect gene promoters with daily active enhancers

**Figure 3.**
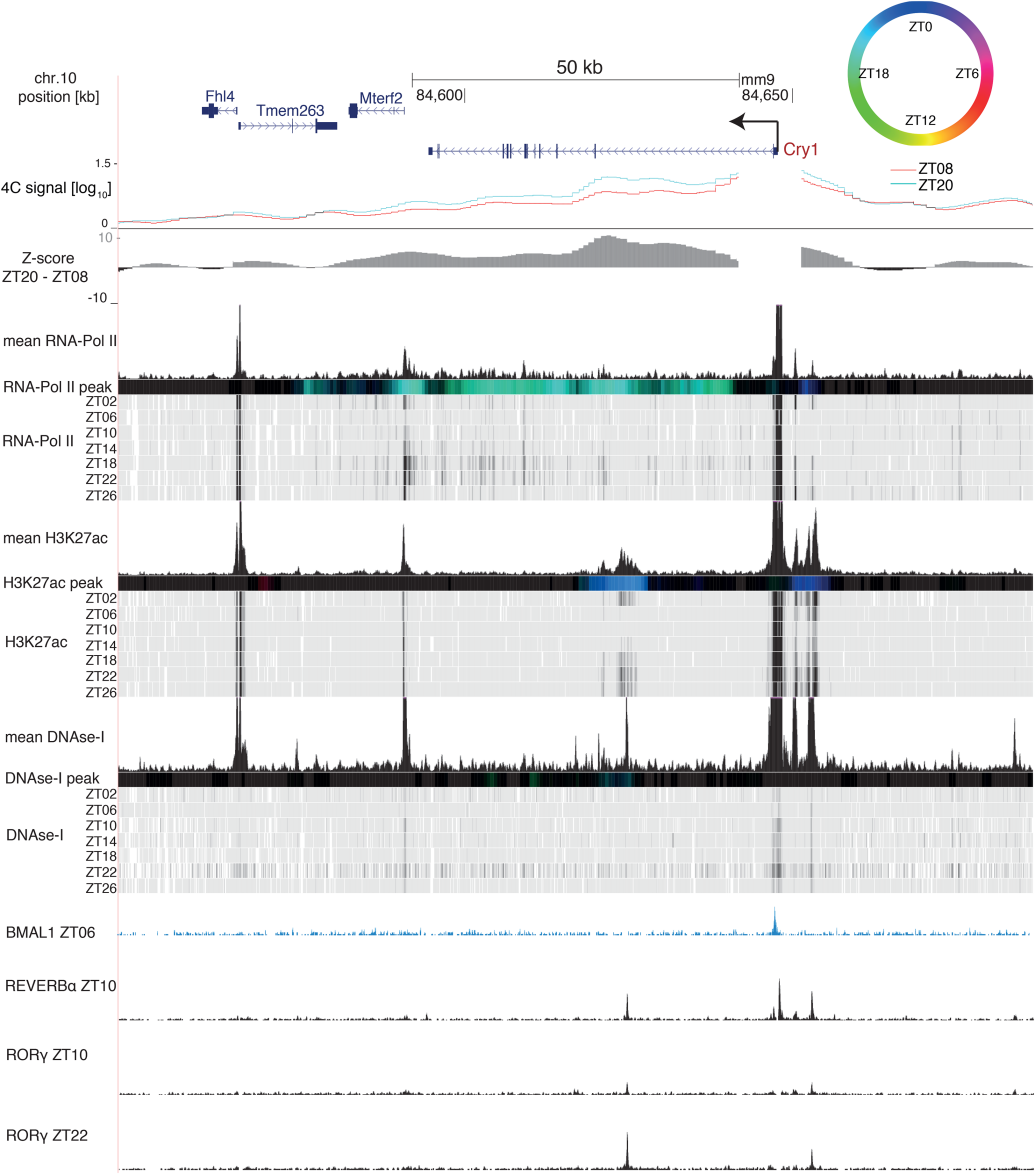
The rhythmic *Cry1* loop connects the promoter with a H3K27ac-marked enhancer. The *Cry1* genomic region containing 4C-seq signals from *Cry1* TSS at ZT08 (red) and ZT20 (blue) and *Z*-score (ZT20–ZT08) in wild-type livers. RNA Pol II loadings (ChIP-seq), H3K27ac mark (ChIP-seq), and DNase-I signal are from Sobel et al. (2017). Temporally averaged signals and temporal signals of each mark are plotted. Colored bars represent peak times according to the color legend at the *top right*; black signifies no rhythm (Materials and Methods). BMAL1 ChIP-seq signal is from Rey et al. (2011), and REV-ERBα and RORγ ChIP-seq signals are from Zhang et al. (2015).

**Figure 4.**
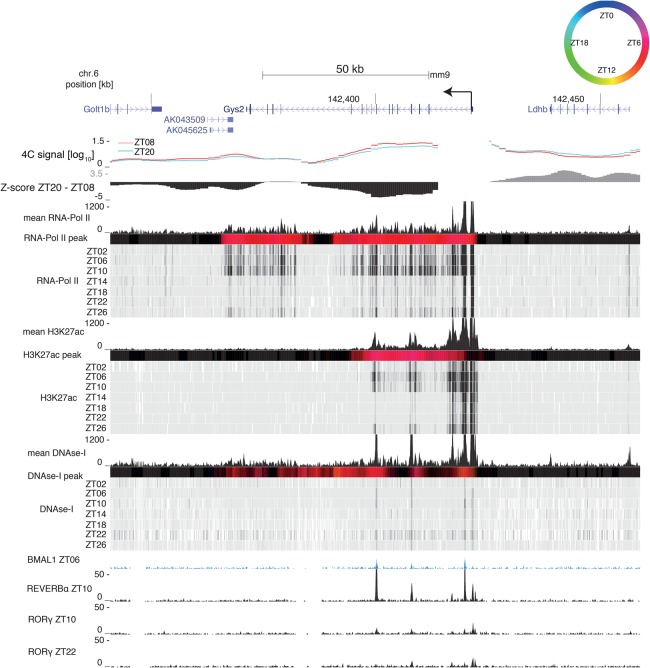
The rhythmic *Gys2* loop connects the promoter with a H3K27ac-marked enhancer. The *Gys2* genomic region containing 4C-seq signals from the *Gys2* TSS at ZT08 (red) and ZT20 (blue) and *Z*-score (ZT20–ZT08) in wild-type livers. RNA Pol II loadings (ChIP-seq), H3K27ac mark (ChIP-seq), and DNase-I signal are from Sobel et al. (2017). Temporally averaged signals and temporal signals of each mark are plotted. Colored bars represent peak times according to the color legend at the *top right*; black signifies no rhythm (Materials and Methods). The BMAL1 ChIP-seq signal is from Rey et al. (2011), and the REV-ERBα and RORγ ChIP-seq signals are from Zhang et al. (2015).

To characterize the interacting genomic regions, we integrated temporal data on DNase-I hypersensitivity sites (DHSs) with ChIP-seq (chromatin immunoprecipitation [ChIP] combined with high-throughput sequencing) data for RNA polymerase II (Pol II), the activity-related chromatin mark H3K27ac (Sobel et al. 2017), and rhythmically active TFs (Rey et al. 2011; Zhang et al. 2015). This allowed us to assess whether the rhythms in DNA contacts coincided with rhythms in activity-related chromatin marks. For *Cry1*, RNA Pol II and H3K27ac signals peaked near ZT20 (Fig. 3), while, for *Gys2*, they peaked near ZT08 (Fig. 4). However, while RNA Pol II signals extended throughout the gene bodies, H3K27ac signals were spatially confined around the largest differential contact precisely at sites marked with DHSs. Furthermore, both the 26-kb downstream intronic site and the 7-kb upstream site of the *Cry1* TSS contained a RORE-responsive element (RRE) and were bound by the circadian TFs REV-ERBα and RORγ (Fig. 3; Supplemental Table S4; Zhang et al. 2015). In mouse fibroblasts, the intronic RRE is required for proper timing of *Cry1* expression (Ukai-Tadenuma et al. 2011). The interacting *Gys2* exon 8 site was bound by the clock regulator BMAL1 at ZT06 (Rey et al. 2011) and by REV-ERBα at ZT10 (Fig. 4). This indicated that DNA contacts connected local rhythmically active enhancer elements with the promoters of *Cry1* and *Gys2*.

### Deleting the *Cry1* intronic enhancer in mice shortens the circadian locomotor period

**Figure 5.**
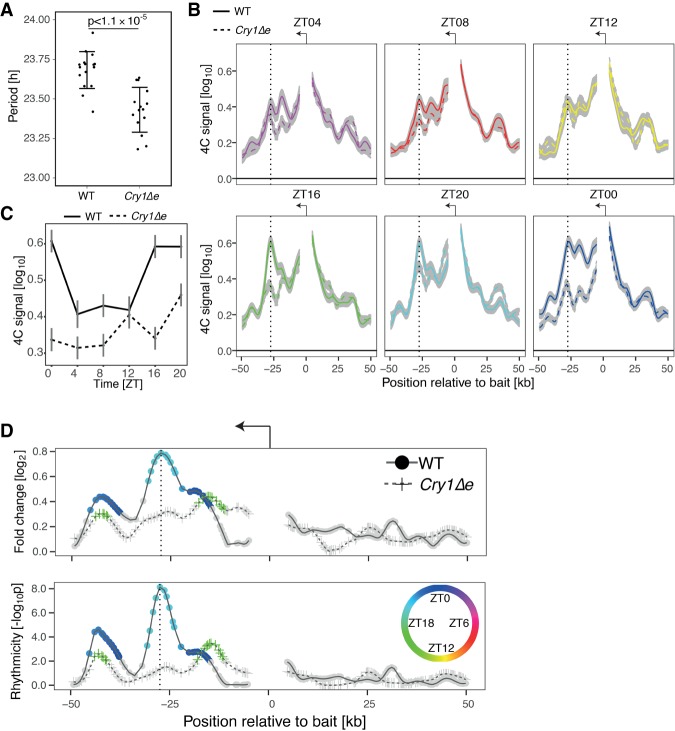
Deleting the *Cry1* intronic enhancer in mice shortens the period of the clock and disrupts oscillations in *Cry1* promoter–enhancer contact frequencies. (*A*) The circadian period of spontaneous locomotor activity is significantly different between *Cry1Δe* and wild-type littermates. The mean period and standard deviation were calculated from 16 wild-type and 15 *Cry1Δe* littermates. *P* = 1.1 × 10^−5^, *t*-test. (*B*) 4C-seq signal for *Cry1* TSS bait over time in livers (LWMR summarizes *n* = 3 animals per group; gray shade shows ±standard error) in wild-type versus *Cry1Δe* littermates. Vertical lines show the +26-kb intronic enhancer. (*C*) 4C-seq signal over time adjacent to the intronic enhancer. (*D*) log_2_ fold change and −log_10_(*p*) from rhythmicity analysis of 4C-seq signal over time. *P* < 10^−8^ at peak, LWMR, χ^2^ test. Fragments with *P*< 0.01 are colored by time of peak contact frequency (color legend is shown at the *right*).

To study the function of the rhythmic chromatin interactions, we generated a mouse strain (*Cry1Δe*) with a 300-base-pair (bp) deletion covering the *Cry1* intronic enhancer (Supplemental Fig. S5A,B). We measured spontaneous locomotor activity in constant darkness and observed that *Cry1Δe* animals had an endogenous circadian period that was significantly shorter (*P* < 1.1 × 10^−5^, *t*-test) by 15 min compared with wild-type littermates (Fig. 5A; Supplemental Fig. S5C,D). Such period shortening is in the range of classic short period core clock mutants such as *Per1* (Cermakian et al. 2001) and *Clock* (Debruyne et al. 2006). As *Cry1* loss of function shortens the circadian period by 1.2-h (van der Horst et al. 1999), our noncoding DNA deletion suggests a *Cry1* hypomorph.

### Expression of *Cry1*, clock, and clock output genes is perturbed in *Cry1Δe*

To investigate the link between the deletion, promoter–enhancer looping, and *Cry1* expression in livers and kidneys, we first generated temporal RNA sequencing (RNA-seq) data in *Cry1Δe* and wild-type littermates under an entraining light–dark cycle. The transcriptomes in *Cry1Δe* and wild-type littermates were comparable overall in both tissues (Supplemental Fig. S6A). While *Cry1* mRNA levels remained rhythmic in both genotypes, likely driven by further regulatory sites (e.g., the TSS and −7-kb sites), the peak expression at ZT20 was significantly reduced by 27% in the livers (15% in the kidneys) of *Cry1Δe* animals compared with wild type (Supplemental Fig. S6B). Quantifying the intronic reads as a proxy for transcription showed that *Cry1* transcription was also phase-advanced in *Cry1Δe* animals (Supplemental Fig. S6C). Moreover, CRY1 protein abundance in the liver was lower in *Cry1Δe* compared with wild type, consistent with a reduction in mRNA levels (Supplemental Fig. S6D,E).

As is known in chronobiology, entraining a short period circadian oscillator by an external light–dark cycle leads to a phase advance of internal timing markers (Aschoff and Pohl 1978). This prediction was confirmed in the transcriptome data. Indeed, core clock and clock-controlled genes (Supplemental Table S5) were phase-advanced by, on average, 30 min in the livers of *Cry1Δe* animals compared with wild type (*P* < 0.01 binomial test) (Supplemental Fig. S6F), with *Cry1* showing the largest phase advance (*P* = 0.011 for livers; *P* = 0.047 for kidneys, bootstrap test) (Supplemental Fig. S6B).

### The *Cry1Δe* mutation disrupts rhythmic chromatin topology

Next, we explored the dynamics of chromatin topology along the 24-h cycle in liver sampled every 4 h in wild type and *Cry1Δe* (*n* = 3 per time point). First, we confirmed oscillatory chromatin interactions in *Gys2* in wild type*.* Indeed, the *Gys2* promoter rhythmically recruited the +21-kb enhancer, peaking near ZT08 in both the TSS bait and exon 8 bait (*P* < 10^−6^ at the peak harmonic regression) (Supplemental Fig. S7A–C). As negative control, the *Hoxd4* bait measured around the clock did not show oscillatory contacts (Supplemental Fig. S7D). For *Cry1* wild type, the frequency of contacts between the promoter and the +26-kb enhancer significantly oscillated, peaking near ZT20 (*P* < 10^−8^ at the peak) (Fig. 5B–D; Supplemental Fig. S8A,B). In contrast, in *Cry1Δe*, the contact frequencies in this region were lower at all time points compared with wild type, and the oscillation was compromised (Fig. 5B–D; Supplemental Fig. S8A,B). Finally, we also estimated chromatin contacts for a bait placed at the −7-kb upstream enhancer (Fig. 1B, bottom tracks, vertical dotted line at the right), showing oscillation in contact frequency peaking around ZT20 with the +26-kb intronic enhancer in wild type but nonrhythmic and overall lower contact frequency in *Cry1Δe* (Supplemental Fig. S8C,D). Decreased contact frequency in *Cry1Δe* mice indicates that the RRE-containing 300-bp fragment drives the promoter–enhancer loop.

Overall, these data demonstrate robust rhythmic chromatin topology for *Cry1* and *Gys2*, where the frequency of enhancer–promoter contacts is modulated with time of day. Furthermore, deleting a localized noncoding DNA enhancer element (300 bp) in the *Cry1* gene could disrupt such rhythms.

### The *Cry1* intronic enhancer modulates transcriptional burst frequency

**Figure 6.**
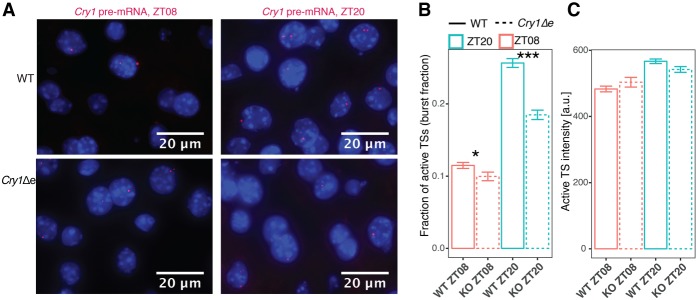
The oscillatory *Cry1* promoter–enhancer loop modulates *Cry1* transcriptional bursting. (*A*) smRNA-FISH against *Cry1* pre-mRNA in the livers of wild-type (*top*) and *Cry1Δe* (*bottom*) animals at ZT08 (*left*) and ZT20 (*right*). Burst fractions (*B*) and burst intensities (*C*) measured from images of smRNA-FISH performed against *Cry1* pre-mRNA in *Cry1Δe* (dashed) and wild-type (solid) livers at ZT08 (red) and ZT20 (blue). Burst fraction is the number of active transcription sites in each nucleus divided by the ploidy. (*B*,*C*) Shown are the means and standard errors over nuclei collected and pooled from two animals in each of the four conditions (individual animals are analyzed in Supplemental Fig. S9C,D). *n* = 2191 wild-type ZT08 nuclei; *n* = 983 *Cry1Δe* ZT08 nuclei; *n* = 2150 wild-type ZT20 nuclei; *n* = 1473 *Cry1Δe* ZT20 nuclei. In *B*, (*) *P* < 0.05; (***) *P* < 0.001, *t*-test. In *C*, differences between genotypes are not significant.

To analyze whether the *Cry1* intronic enhancer modulates transcription, we estimated transcriptional parameters by smRNA-FISH against *Cry1* pre-mRNA in the livers of wild-type and *Cry1Δe* animals at ZT08 and ZT20 (Fig. 6A). Mammalian promoters are irregularly transcribed (transcriptional bursting), as characterized by the burst size and burst frequency (Suter et al. 2011; Bahar Halpern et al. 2015). Taking into account the ploidy of liver nuclei (Supplemental Fig. S9A–D), smRNA-FISH showed that *Cry1* burst fraction (fraction of active transcription sites in each nucleus, which is proportional to the burst frequency per allele) was 2.2-fold higher at ZT20 compared with ZT08 in wild type (Fig. 6B). Importantly, the burst fraction was reduced by 28% in *Cry1Δe* animals at ZT20 (Fig. 6B). In contrast, the burst intensity (proportional to the burst size) was similar in all conditions (Fig. 6C). Thus, the lowered *Cry1* mRNA levels in *Cry1Δe* at ZT20 can be quantitatively explained by the reduced burst fraction. In sum, dynamic enhancer loops modulate transcriptional bursting in mammalian tissues (Bartman et al. 2016; Fukaya et al. 2016); in particular, rhythmic DNA loops involving clock enhancers control burst frequency while maintaining burst size.

## Discussion

In animals, developmental transitions occurring on the time scales of days have been shown to involve remodeled DNA contacts and promoter–enhancer loop formation (Noordermeer et al. 2014). While such dynamics are typically irreversible, we here discovered that chromatin topology in mouse tissues can be locally (100 kb, in *cis*) plastic, exhibiting temporal dynamics that are regulated by daily time and the circadian oscillator and thus recur within a 24-h period. While previous work in cell culture reported dynamic chromatin contacts on larger genomic scales, notably between the *Dbp* gene and DNA regions on *trans* chromosomes (Aguilar-Arnal et al. 2013), the genes analyzed here did not show rhythmic chromatin interactions on such scales. We then showed genetically that these rhythmic DNA contacts depend on the clock protein BMAL1 and, in the case of *Cry1*, a 300-bp intronic RRE-containing enhancer sequence.

How is BMAL1 involved in the formation of these dynamic loops? In the case of *Gys2* in *Bmal1* knockout mice*,* the loop is constitutively open, and *Gys2* mRNA expression is constitutively low. Combined with the binding of BMAL1 at the looping site, these data strongly argue for a direct involvement of BMAL1. For *Cry1*, the activator RORγ and the repressor REV-ERBα bind to the *Cry1* intronic enhancer at the expected peak (ZT20) and trough (ZT08) activities, as is typical of functional RREs*.* We note that while the expression of the RRE-binding repressors *Rev-Erb*α/β is low in *Bmal1* knockout, the corresponding activator *Ror*γ is constitutively high (Atger et al. 2015). Therefore, the constitutively closed *Cry1* loop in the *Bmal1* knockout most likely reflects an indirect effect via perturbed REV-ERB and ROR activities. This is further corroborated by the constitutively open state of the *Cry1* promoter–enhancer loop in *Cry1Δe* mice, showing chromatin interactions that are constantly below wild-type trough levels, indicating that loop-promoting factors (for example, RORs) act within the 300-bp element. Therefore, our data suggest a canonical mechanism of enhancer–promoter looping by which sequence-specific TFs help recruit transcription complexes, which facilitate the function of Pol II at core promoters (Levine and Tjian 2003).

To investigate the effects of the dynamic looping on transcriptional parameters, we complemented bulk 4C-seq and RNA-seq experiments with single-molecule transcript analysis in situ, which revealed that the abolished rhythmic chromatin contact in *Cry1Δe* mice reduced *Cry1* transcriptional burst frequency. These results in mammalian tissues contribute to our current understanding of how enhancer loops modulate transcriptional bursting (Bartman et al. 2016; Fukaya et al. 2016). In particular, we showed that rhythmically active clock enhancers can increase burst frequency while not changing burst size.

The ablation of the *Cry1* noncoding regulatory element even led to a short period phenotype in locomotor activity. While noncoding genetic variation in humans has been associated recently with circadian clock-related and sleep phenotypes (Allebrandt et al. 2010; Hu et al. 2016), no demonstration of such variation on circadian transcription or behavior has yet been provided. Indeed, previously characterized mutations impacting mammalian circadian behavior have concerned protein-coding regions (Vitaterna et al. 1994; Toh et al. 2001). Here, we provided evidence that noncoding regulatory elements within the core circadian regulatory network can drive dynamic promoter–enhancer looping, modulate temporal transcription, and regulate circadian locomotor behavior.

## Materials and methods

### Animal and ethics statement

All animal care and handling were performed according to Canton de Vaud laws for animal protection (authorization VD2801 [Frédéric Gachon] and VD3109 [Félix Naef]). All experiments were performed on males between 8 and 10 wk old. *Bmal1* knockout animals were described previously in Jouffe et al. (2013).

### Mouse genome editing by direct knockout using CRISPR–Cas9

Px-330 plasmids targeting upstream of and downstream from the *Cry1* intron1 regulatory region were injected into pronuclei and then transplanted into B6D2F1 pseudopregnant mice at the Ecole Polytechnique Fédérale de Lausanne (EPFL) Transgenic Core Facility (http://tcf.epfl.ch). Pups from the first generation (F0) were then screened for the deletion using the PCR primers indicated in Supplemental Table S2. F0 animals of interest were backcrossed on C57/BL6J wild-type mice, and F1 animals were screened for transmission of the mutation. Heterozygous animals were crossed together to obtain all genotypes of interest. The Ethical Committee of the State of Vaud Veterinary Office, Switzerland, approved all experiments.

### Nucleus purification and fixation

Immediately after sacrifice, 5 mL of 1× PBS was perfused through the spleen to flush blood from the liver. Livers and kidneys from individual animals were homogenized and fixed in 4 mL of 1× PBS, including 1.5% formaldehyde, for 10 min at room temperature. The cross-linking reaction was stopped by adding 25 mL of ice-cold stop reaction buffer (2.2 M sucrose, 150 mM glycine, 10 mM HEPES at pH 7.6, 15 mM KCl, 2 mM EDTA, 0.15 mM spermine, 0.5 mM spermidine, 0.5 mM DTT, 0.5 mM PMSF) to the homogenates and was kept for 5 min on ice. Homogenates were then loaded on top of 10 mL of cushion buffer (2.05 M sucrose, 10% glycerol, 125 mM glycine, 10 mM HEPES at pH 7.6,15 mM KCl, 2 mM EDTA, 0.15 mM spermine, 0.5 mM spermidine, 0.5 mM DTT, 0.5 mM PMSF) and centrifuged at 10^5^*g* for 45 min at 4°C. Nuclei were washed twice in 1× PBS and immediately frozen.

### 4C-seq

#### 4C template preparation

4C templates were prepared as in Gheldof et al. (2012). Nuclei were resuspended in 1 mL of a buffer containing 10 mM Tris-HCL (pH 8.0), 10 mM NaCl, 0.2% NP-40, and 1× protease inhibitor cocktail (Complete Mini EDTA-free protease inhibitor cocktail; Sigma-Aldrich); kept for 15 min on ice; and washed twice with 1× DpnII buffer (New England Biolabs). Thirty million nuclei were resuspended in 1× DpnII buffer (New England Biolabs) containing 0.1% SDS and incubated for 10 min at 65°C. Triton X-100 was added to 1% final concentration. Chromatin was digested overnight with 400 U of DpnII (New England Biolabs) at 37°C with shaking. After heat inactivation, digestion efficiency was evaluated by both DNA visualization on agarose gels and quantitative PCR using primer pairs covering multiple restriction sites. Chromatin was then ligated with 3000 U of T4 DNA ligase (New England Biolabs) in an 8-mL final volume for 4 h at 16°C plus 1 h at room temperature. The cross-linking reaction was reverted by the addition of 50 µL of 10 mg/mL proteinase K and incubation overnight at 65°C. DNA was purified by multiple phenol/chloroform extractions, resuspended in TE buffer (pH 8.0) containing RNase A, and incubated for 30 min at 37°C. Ligation efficiency was evaluated by loading DNA on an agarose gel. Libraries were digested with 1 U of NlaIII per microgram of template (New England Biolabs) overnight at 37°C, and digestion was controlled by visualization on an agarose gel. After heat inactivation, digested products were ligated with 2000 U of T4 DNA ligase (New England Biolabs) for 4 h at 16°C in a 14-mL final volume. Circularized products were purified and resuspended in TE buffer (pH 8.0). 4C templates were prepared in four biological replicates in wild-type mouse livers and kidneys and three biological replicates in the livers of *Bmal1* knockout and *Cry1Δe* and wild-type littermates (Supplemental Table S1).

#### Inverse PCR and sequencing in wild-type and Bmal1 knockout mouse livers and kidneys

Six-hundred nanograms of 4C template was used for PCR amplification using Sigma-Aldrich long-template PCR system with bait-specific inverse primers conjugated to Illumina sequencing adaptors (primer sequences are in Supplemental Table S3) in a final volume of 50 µL in the following PCR program: 2 min at 94°C followed by 30 cycles of 15 sec at 94°C, 1 min at 55°C, and 3 min at 68°C and a final extension of 7 min at 68°C. PCR were performed in parallel reactions with 6 × 100 ng of template for each sample. PCR products were purified with the AMPure XP beads system (Beckman Coulter), and amplification profiles were analyzed by fragment analyzer and then sequenced on Illumina HiSeq 2000 machines using single-end 100-bp read length.

#### Inverse PCR and sequencing in the livers of Cry1Δe and wild-type littermates

Six-hundred nanograms of 4C template was used for PCR amplification using Sigma-Aldrich long-template PCR system with two-step PCR system from Illumina. Bait-specific inverse primers conjugated to Illumina sequencing adaptors (primer sequences are in the Supplemental Table S3) were used in a first PCR reaction in a final volume of 50 µL with the following program: 2 min at 94°C followed by 20 cycles of 15 sec at 94°C, 1 min at 55°C, and 3 min at 68°C and a final extension of 7 min at 68°C. PCRs were performed in parallel reactions with 6 × 100 ng of template for each sample. PCR products were purified with the AMPure XP beads system (Beckman Coulter). Purified products were pooled and used as the template of a second PCR reaction with Nextera XT index kit version2 primers (FC-131-2004) in a final volume of 50 µL with the following program: 2 min at 94°C followed by 10 cycles of 15 sec at 94°C, 1 min at 55°C, and 3 min at 68°C and a final extension of 7 min at 68°C. PCR products were purified with the AMPure XP beads system (Beckman Coulter) and then sequenced on NextSeq 500 machines using single-end 75-bp read length.

### 4C-seq analysis

#### Preprocessing computational methods

Demultiplexed Fastq files were mapped to the mouse genome (mm9) using Bowtie2 with default HTSstation parameters (http://htsstation.epfl.ch). Since each restriction fragment contained two mapping sites (two ends of the fragment), the fragment score was computed as the average of the number of reads per mapping site.

#### Quality control of 4C-seq data

Samples with ≥75% of restriction fragments without any counts in a window of ±1 Mb upstream of and downstream from each bait were not analyzed (Supplemental Table S1). The first five fragments upstream of and downstream from the bait (10 total) were not considered in the analysis because they mostly contained partially digested and self-ligated products.

#### Normalization and LWMR

We follow a method developed recently in Yeung et al. (2018) with minor modifications. Briefly, raw read counts for each sample were library size-rescaled by the normalized sum of the read counts on the *cis* chromosome (excluding 10 restriction fragments around the bait). To control the variability of low signals, in subsequent analyses, the fragment counts *c* in each sample were log transformed using the variable

$$Y=\log_{10}\left(\frac{c}{P}+1\right),$$

with *P* = 500. A weighted linear model was then fit locally using a Gaussian window (σ_*G*_ = 2500 bp) centered on the fragment of interest. For each position, nearby 4C-seq signals (*Y*) were modeled with fragment effects *a*_*i*_ and condition effects *b*_*j*_ (which can be time, tissue, or genotype). In LWMR, these parameters were estimated by minimizing the weighted sum *S* of squared residuals across replicates *r*: *S* = argmin_*a,b*_Σ_*i,j,r*_W_*i,j*_(*Y*_*i,j,r*_ − *a*_*i*_ − *b*_*j*_)^2^, with weights *W*_*i,j*_ defined as *W*_*i,j*_ = *w*_*g,i*_ × *w*_*s,j*_, where *w*_*g,i*_ is the Gaussian smoothing kernel at position *i*, and *w*_*s,j*_ is a condition weight based on the number of samples with nonzero counts on fragment *i*. Specifically, we used *w*_*s*_ = 0.5, 1.5, 2.5, 3.5, or 4.5 for fragments with zero, one, two, three, or four replicates showing nonzero counts, which down-weighs positions with high dropout rates. To estimate the statistical significance for differential contacts (for example, ZT20 vs. ZT08), we propagated the estimated uncertainty (standard errors for locally weighted regression) in the corresponding *b* values to calculate *Z*-scores and used regularized *t* statistics with *n* – *p* degrees of freedom (DOF; *n* is the number of data points within window, and *p* is the number of parameters). For the analysis of 24-h rhythmicity in contacts (weighted harmonic regression), we proceeded analogously by propagating the uncertainty in the *b*s for the six time points to that in the squared 24-h Fourier coefficient and used the χ^2^ test with two DOF (owing to the real and imaginary parts). For each set of samples, we computed the regularized residual variance as

$${\tilde{\sigma }}^{2}={\hat{\sigma }}^{2}+\sigma_{\min}^{2}\exp\left(-\frac{\bar{b}}{b_{s}}\right),$$

with ${\hat{\sigma }}^{2}$ as the estimator of the squared residuals, $\bar{b}$ as the estimated signal across samples, and *b*_*s*_ = log_10_(2). $\sigma_{\min}^{2}$ prevents artificially small variance from positions of high dropout rates and is estimated from the distribution of ${\tilde{\sigma }}^{2}$ across all fragments. σ_min_ ranges from 0.06 to 0.16 (same units as *Y*), depending on the bait (Supplemental Table S1).

### H3K27ac and RNA Pol II ChIP-seq and DNase-I-seq analysis

Bam files from GSE60578 (Sobel et al. 2017) were analyzed in genomic regions ±1 Mb from the 4C-seq baits. There, read counts were binned in 500-bp intervals and normalized by the library size. The amplitude and phase of the log_2_ read counts of each of the three signals were calculated for each bin after applying a running average of seven bins (three bins upstream, three bins downstream, and one bin in the center) to smooth the signal. Obtained rhythmic amplitudes and phases were compared with differential 4C-seq signals. The rhythmic signal in each bin [phase, amplitude, and −log_10_(*p*)] was mapped to a color using the hue, saturation, and value (HSV) color scheme. Hue *h* was defined by the phase of the oscillation, with blue as ZT0. The saturation *s* was set to 1. The value *v* was set to a color if both amplitude *X*_*a*_ and −log_10_(*p*) *X*_*p*_ were beyond thresholds *k*_*a*_ = 1,*k*_*p*_ = 4.5; otherwise, the color was set to black. To obtain smooth transitions, *v* was calculated using a Hill function with Hill coefficient *n* = 5 and

$$v=\min_{i\in (a,p)}\left(\frac{-\log{\left(x_{i}\right)}^{5}}{k_{i}^{5}-\log{\left(x_{i}\right)}^{5}}\right).$$

For TF-binding site predictions (Supplemental Table S4), we used weight matrices of TFs defined by SwissRegulon (Pachkov et al. 2007; http://swissregulon.unibas.ch/fcgi/sr/downloads).

### RNA-seq in the livers and kidneys of *Cry1Δe* and wild-type littermates

Parts of the livers and kidneys from the animals used for temporal 4C-seq experiments were frozen in liquid nitrogen immediately after sacrifice. Organs were homogenized in 4 M guanidine thiocyanate, 25 mM sodium citrate, 1% β-mercaptoethanol, and 0.2 M sodium acetate. Nucleic acids were extracted with phenol:chloroform:isoamylalcohol, and RNA was precipitated with 4 M LiCl. RNA concentration and purity were measured using nanodrop, and the quality was controlled by fragment analyzer. Poly-A-selected RNA was sequenced on NextSeq 500 machines using single-end 75-bp read length. mRNA levels were quantified using kallisto version 0.42.4 (mm10) (Bray et al. 2016).

### RNA-seq in the livers and kidneys of *Bmal1* knockout and wild-type mice

To complement the mouse liver wild-type and *Bmal1* knockout RNA-seq data (GSE73554), transcriptomes of kidneys from wild-type animals were measured following the same protocol as in Atger et al. (2015). mRNA levels were quantified using the same method as in Atger et al. (2015).

### Circadian period estimation in *Cry1Δe* animals and wild-type littermates

Estimation of the circadian period was performed as in Diessler et al. (2017). Briefly, 8- to 10-wk-old males were single-caged and kept under 12 h/12 h light/dark cycle for 14 d and switched to constant darkness for 21 d. During the 5 wk of the experiment, the locomotor activity was recorded with passive infrared sensors. Data were sampled with 5-min resolution and analyzed using the χ^2^ periodogram function in the ClockLab software (ActiMetrics). Food and water were available ad libitum during the entire experiment.

### Western blotting

Liver cytoplasmic extracts were prepared as described previously (Jouffe et al. 2013). Protein extract concentrations were quantified using a BCA protein assay kit (Thermo Fisher Scientific), and 20 µg of liver protein extract was resolved by SDS-PAGE using standard procedures. Densitometry analyses of the blots were performed using the ImageJ software. Naphtol blue and black staining of the membranes was used as a loading control and served as a reference for normalization of the quantified values. CRY1 antibody (1/500) was from Abcam (ab104736).

### smRNA-FISH on mouse liver sections

Parts of the livers from the same animals used in the 4C-seq and RNA-seq were collected, immediately embedded in O.C.T. compound (Tissue-Tek, Sakura-Finetek USA), and snap-frozen. The RNA-FISH was done on 8-µm cryosections using a RNAscope probe for *Cry1* pre-mRNA (Cry1_intron1*,* catalog no. 500231) according to the manufacturer's instructions for the RNAscope fluorescent multiplex assay (Advanced Cell Diagnostics). Nuclei were counterstained with DAPI, and sections were mounted with ProLong Gold anti-fade mountant (Molecular Probes).

### Microscope image acquisition, quantification, and ploidy assignment

The sections were imaged using a Leica DM5500 wide-field microscope equipped with a CCD camera (DFC 3000) for fluorescence (Leica Microsystem) and a motorized stage. *Z*-stacks were aquired (0.2 µm between each *Z* position, 40 images per frame) with an oil immersion 63× objective. The images were quantified using ImageJ. To detect the fluorescent RNA-FISH spots, a Laplacian filter was applied on a maximal projection, and local maxima were computed. Transcription site fluorescent intenstities (burst size) were quantified on the sum projection of the nine best-focused stacks per image. Total transcription site signals were computed using a mask of 3 × 3 pixels. Nuclei were detected using filters, thresholding, and watershed transformation. Ploidy (2N, 4N, or 8N) was assigned to the nuclei based on their diameter (Bahar Halpern et al. 2015). A four-component Gaussian mixture model was fitted to the diameter distribution (package “mixtools” in R). Nuclei with a probability of >0.7 to belong to one of the three inferred populations with the smallest means were assigned to 2N, 4N, and 8N, respectively. The Gaussian distribution with the largest variance captured outliers in nucleus diameters (>15–18 µm) and were discarded. Burst fraction was calculated as the number of active transcription sites in each nucleus divided by its estimated ploidy, and these fractions were then averaged over the entire populations of nuclei (Fig. 5B,C). For Supplemental Figure S10C, we modeled the number of active transcription sites with genotype-dependent slopes and compared it with a reduced model without a genotype effect (lme4 function in R, likelihood ratio test). For Supplemental Figure S10D, we modeled the mean intensity of intronic dots with genotype-dependent intercepts and compared it with a reduced model with a single intercept.

### Data availability

Raw and processed sequencing data generated from this study (4C-seq and RNA-seq) have been submitted to Gene Expression Omnibus under accession number GSE101423.

## Supplementary Material

Supplemental Material
