# Supplementary material for: Clock-dependent chromatin topology modulates circadian transcription and behavior
Source: Genes Dev. 2018 Mar 1;32(5-6):347–58. doi: 10.1101/gad.312397.118 (PMC5900709; doi:10.1101/gad.312397.118)
Supplement: Supplemental Material [file supp_32_5-6_347__index.html]

Clock-dependent chromatin topology modulates circadian transcription and behavior — Supplemental Material 

# Clock-dependent chromatin topology modulates circadian transcription and behavior

## Supplemental Material

- Supplemental\_Fig\_S1.pdf
- Supplemental\_Fig\_S2.pdf
- Supplemental\_Fig\_S3.pdf
- Supplemental\_Fig\_S4.pdf
- Supplemental\_Fig\_S5\_revised\_rearranged.pdf
- Supplemental\_Fig\_S6\_revised\_rearranged.pdf
- Supplemental\_Fig\_S7\_revised.pdf
- Supplemental\_Fig\_S8\_revised.pdf
- Supplemental\_Fig\_S9\_revised.pdf
- Supplemental\_Table\_S1.xlsx
- Supplemental\_Table\_S2.xlsx
- Supplemental\_Table\_S3.xlsx
- Supplemental\_Table\_S4.bed
- Supplemental\_Table\_S5.xlsx
- Supplemental\_Captions.docx
