# Supplementary figures and images for: Clock-dependent chromatin topology modulates circadian transcription and behavior
Source: Genes Dev. 2018 Mar 1;32(5-6):347–58. doi: 10.1101/gad.312397.118 (PMC5900709; doi:10.1101/gad.312397.118)

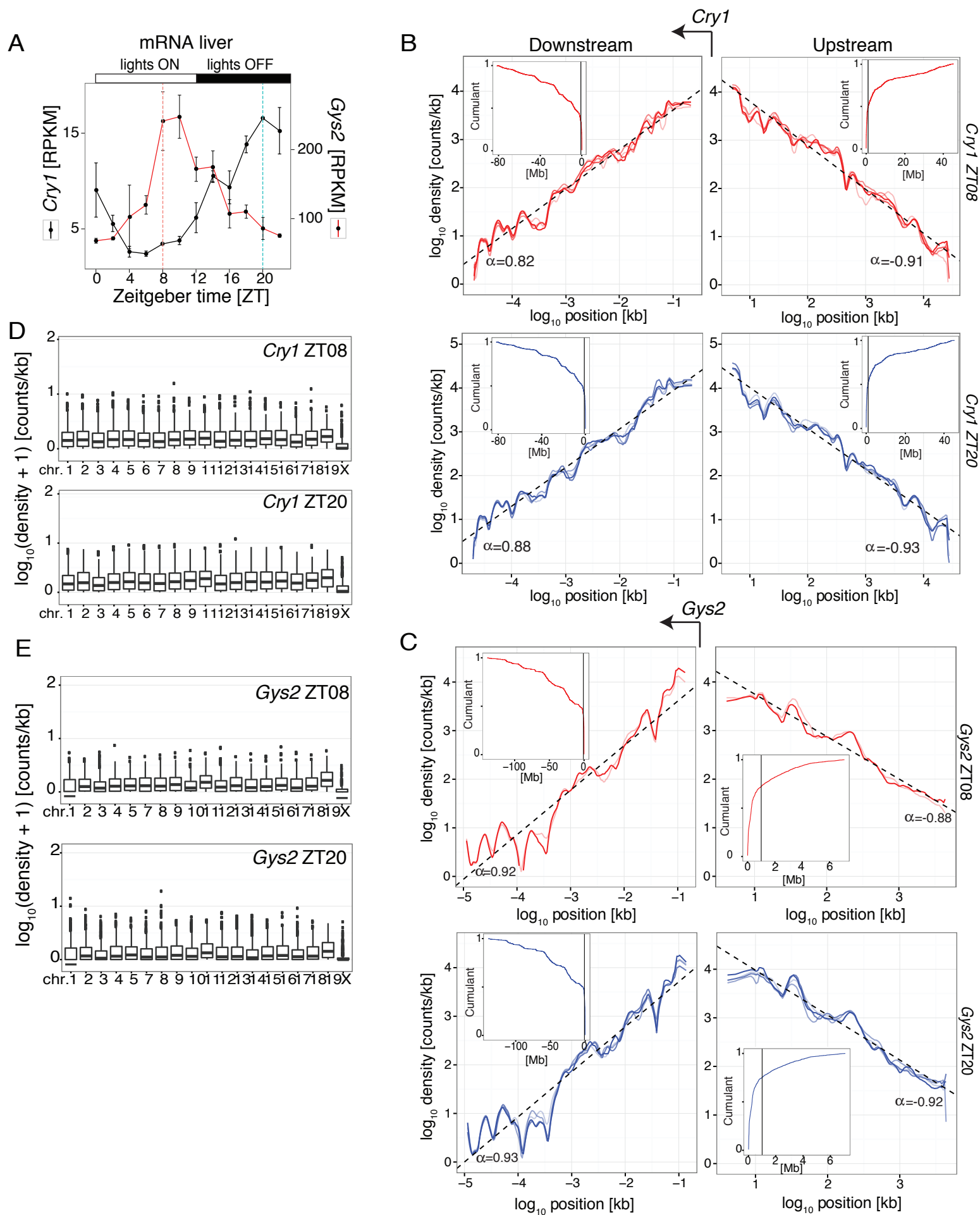

Supplement: Supplemental Material [file supp_gad.312397.118_Supplemental_Fig_S1.pdf]

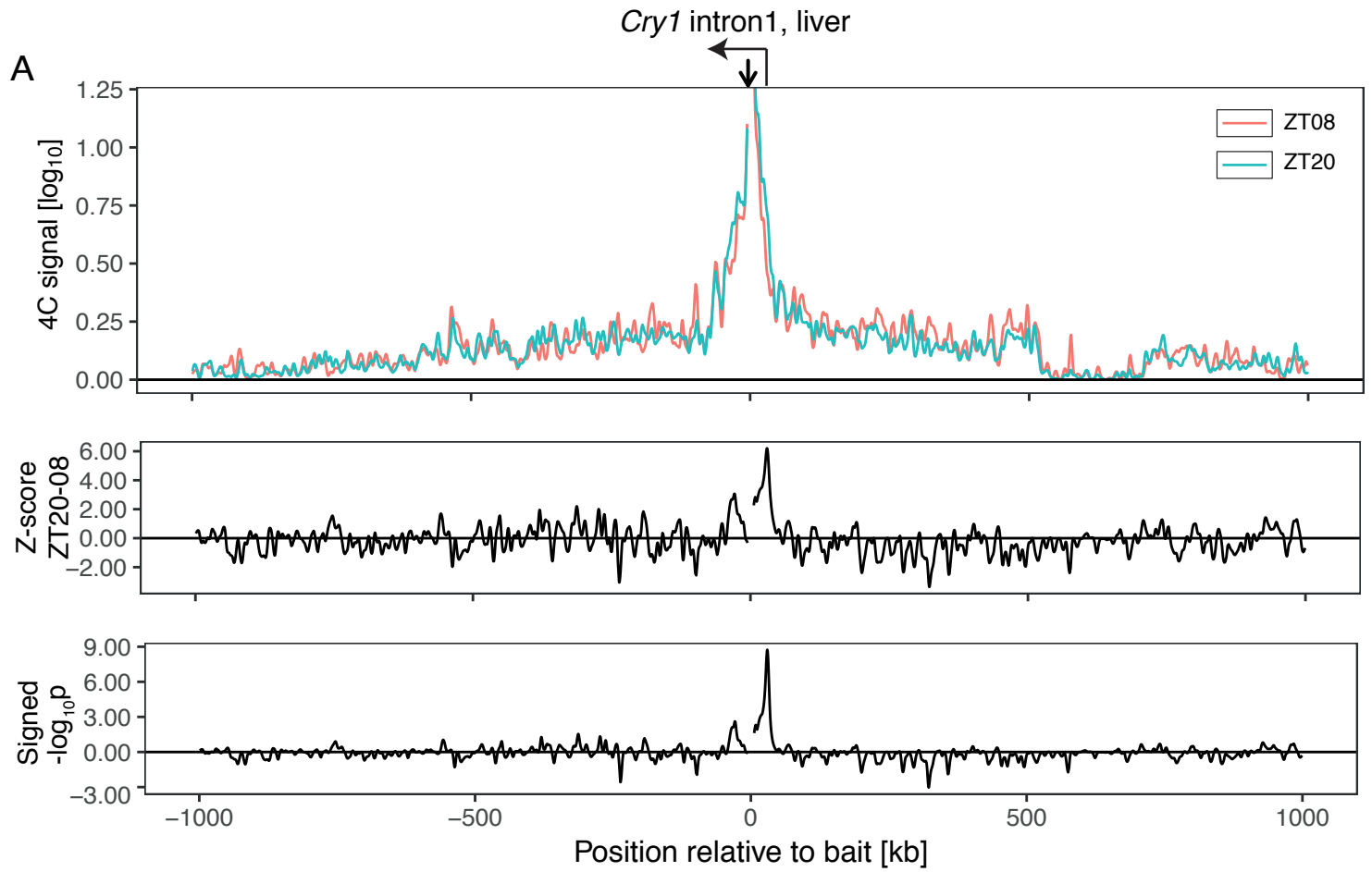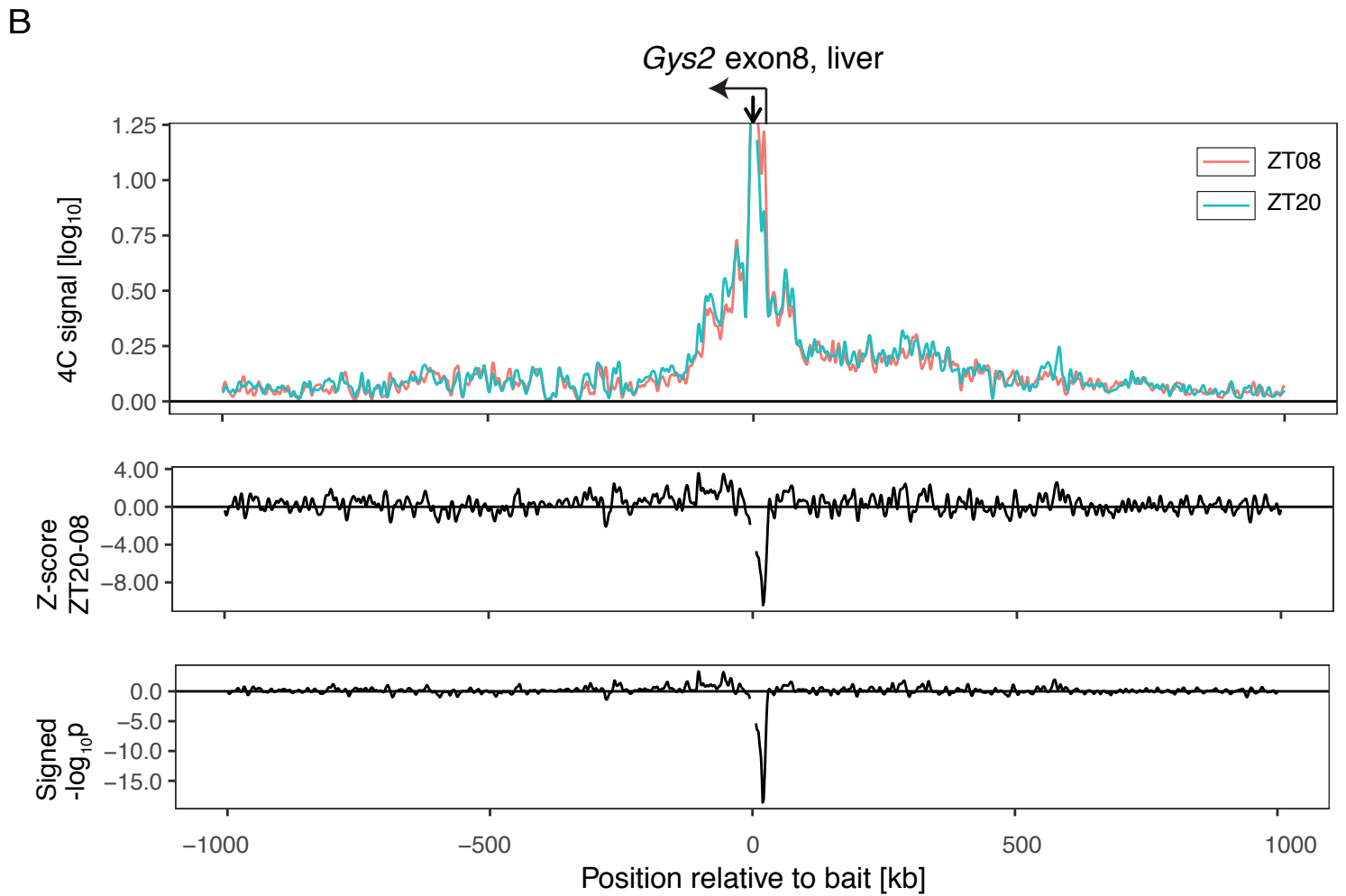

Supplement: Supplemental Material [file supp_gad.312397.118_Supplemental_Fig_S2.pdf]

**A**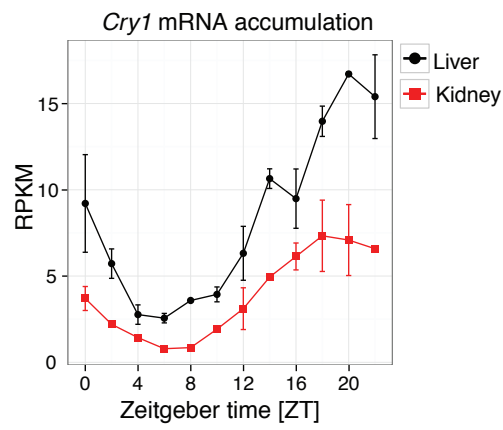**D**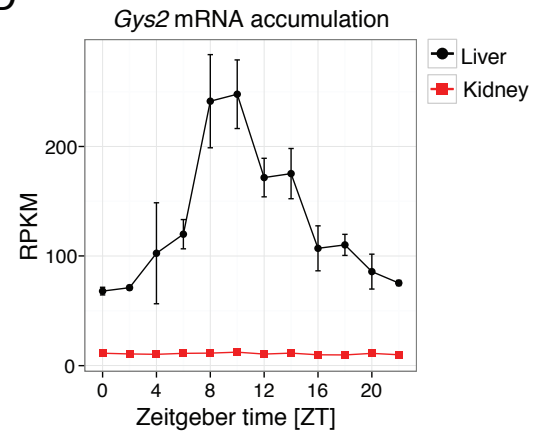**B**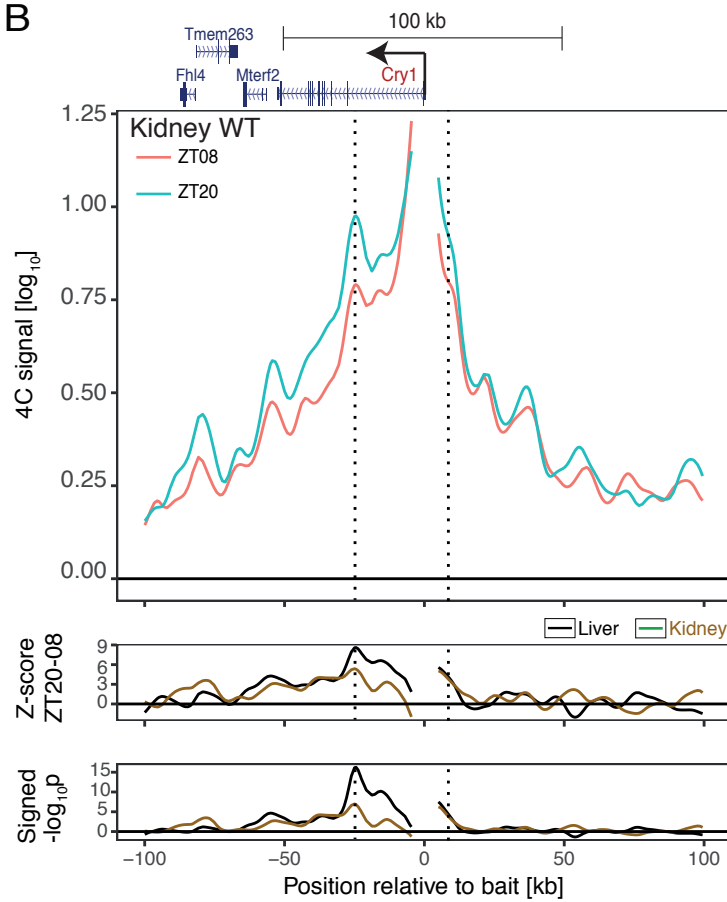**E**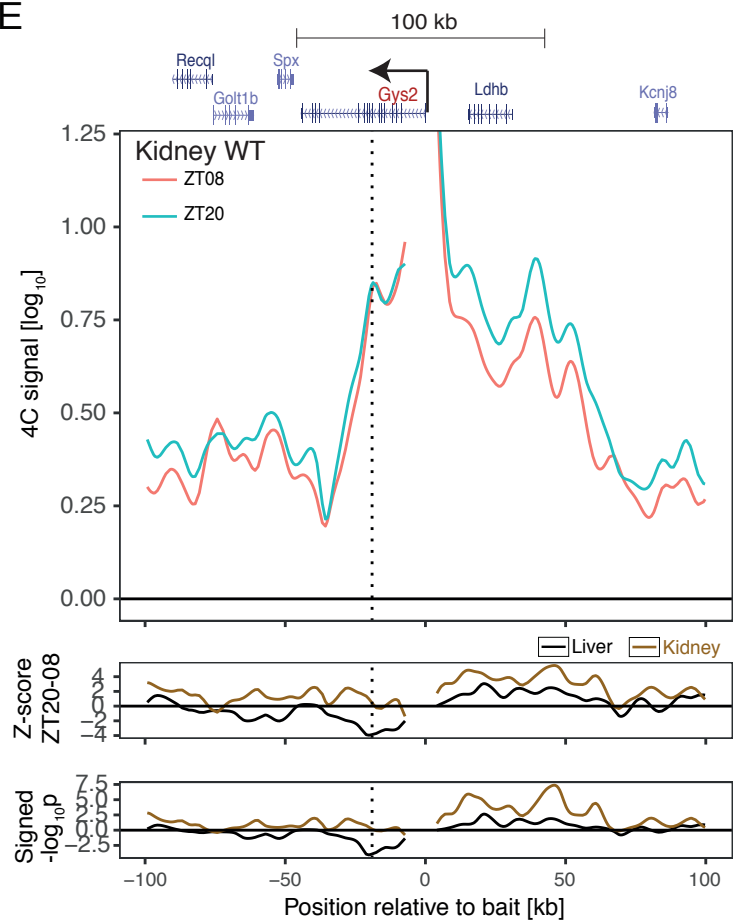**C**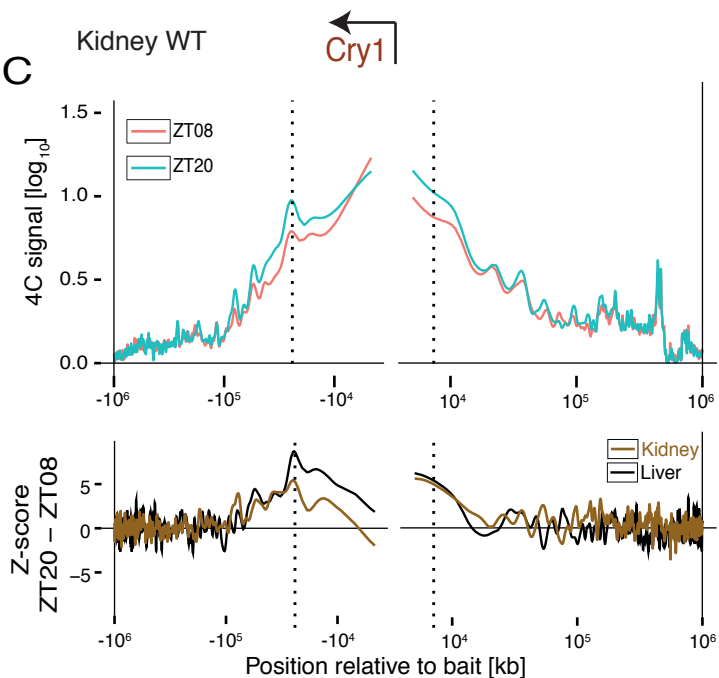**F**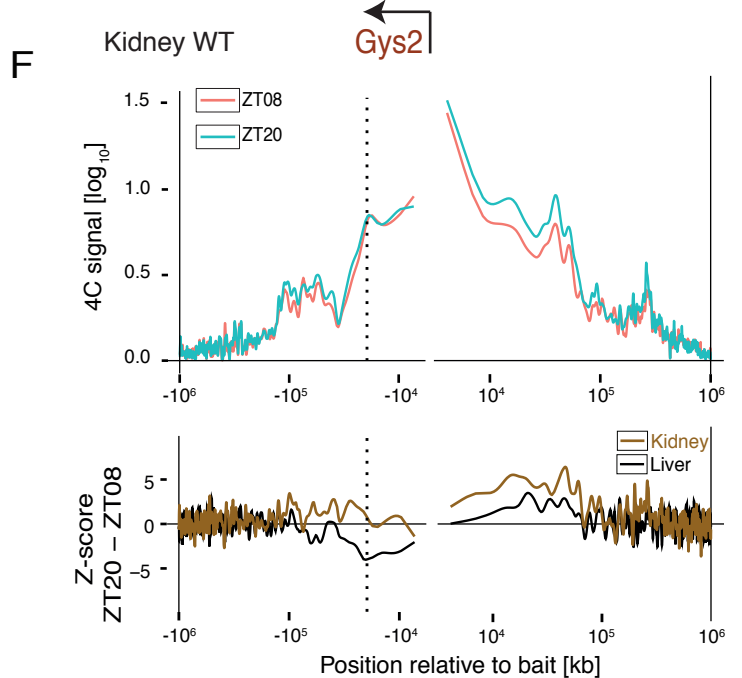

Supplement: Supplemental Material [file supp_gad.312397.118_Supplemental_Fig_S3.pdf]

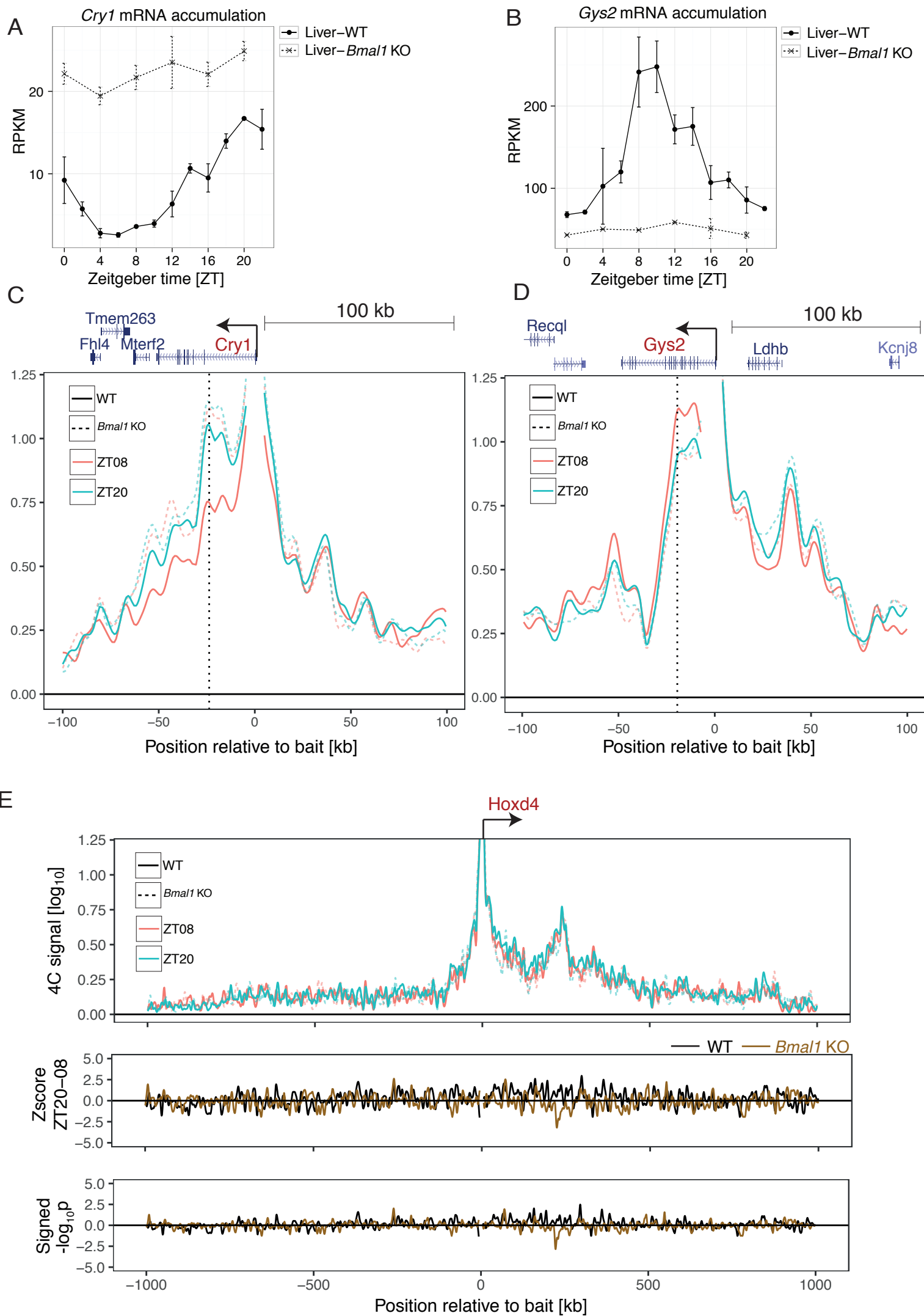

Supplement: Supplemental Material [file supp_gad.312397.118_Supplemental_Fig_S4.pdf]

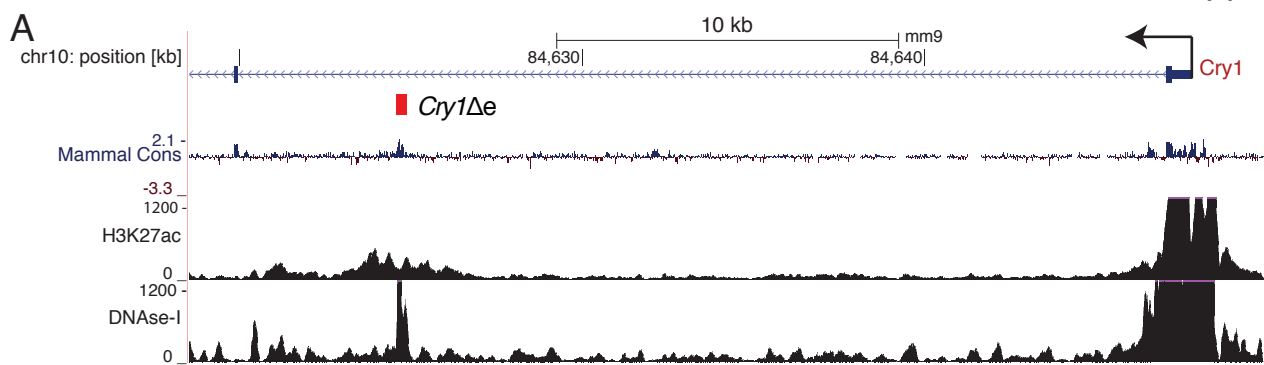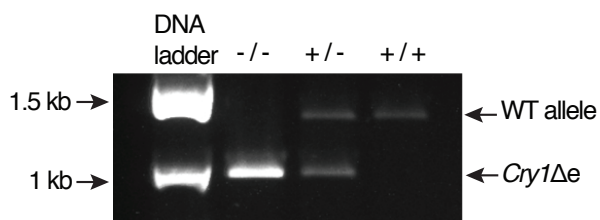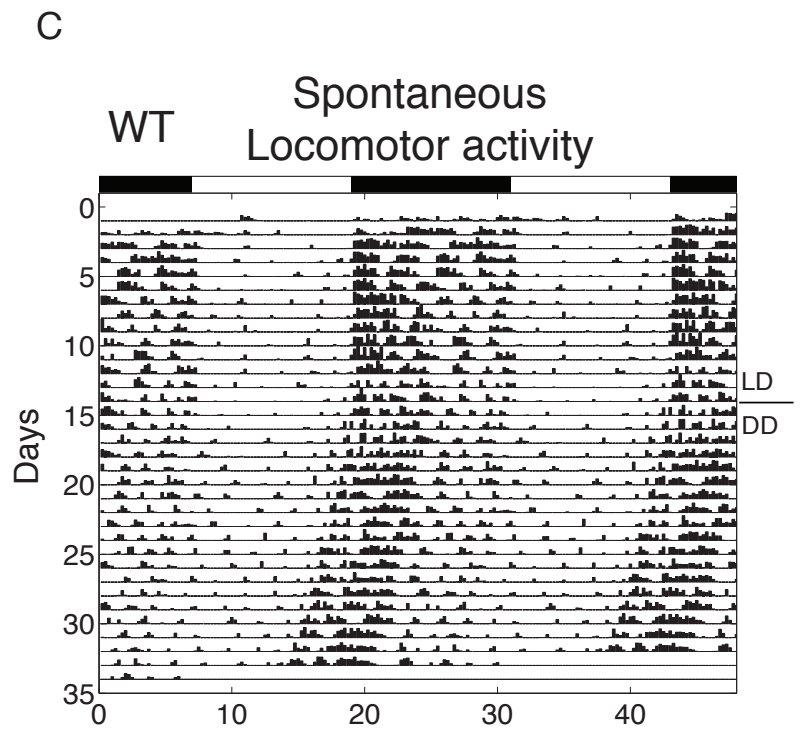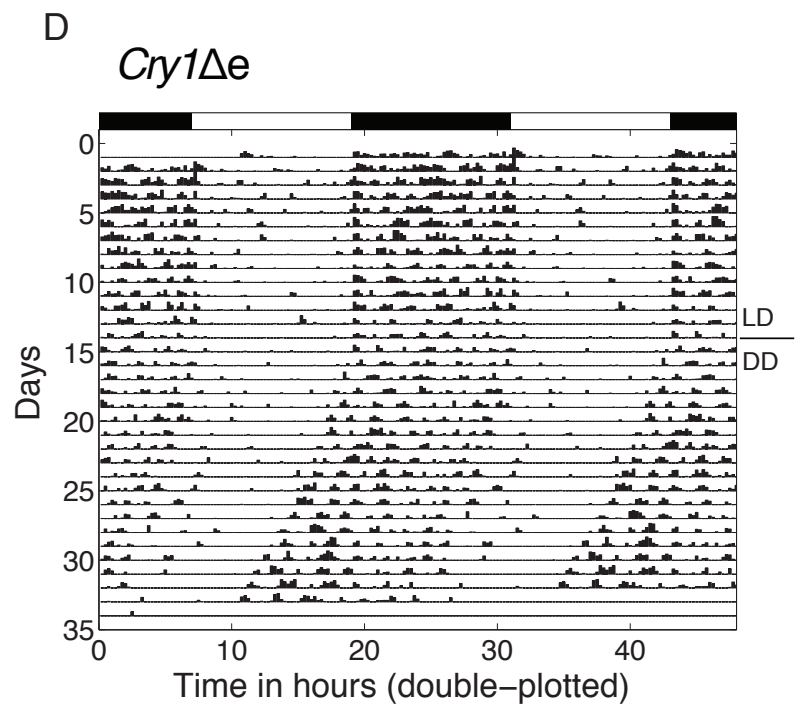

Supplement: Supplemental Material [file supp_gad.312397.118_Supplemental_Fig_S5_revised_rearranged.pdf]

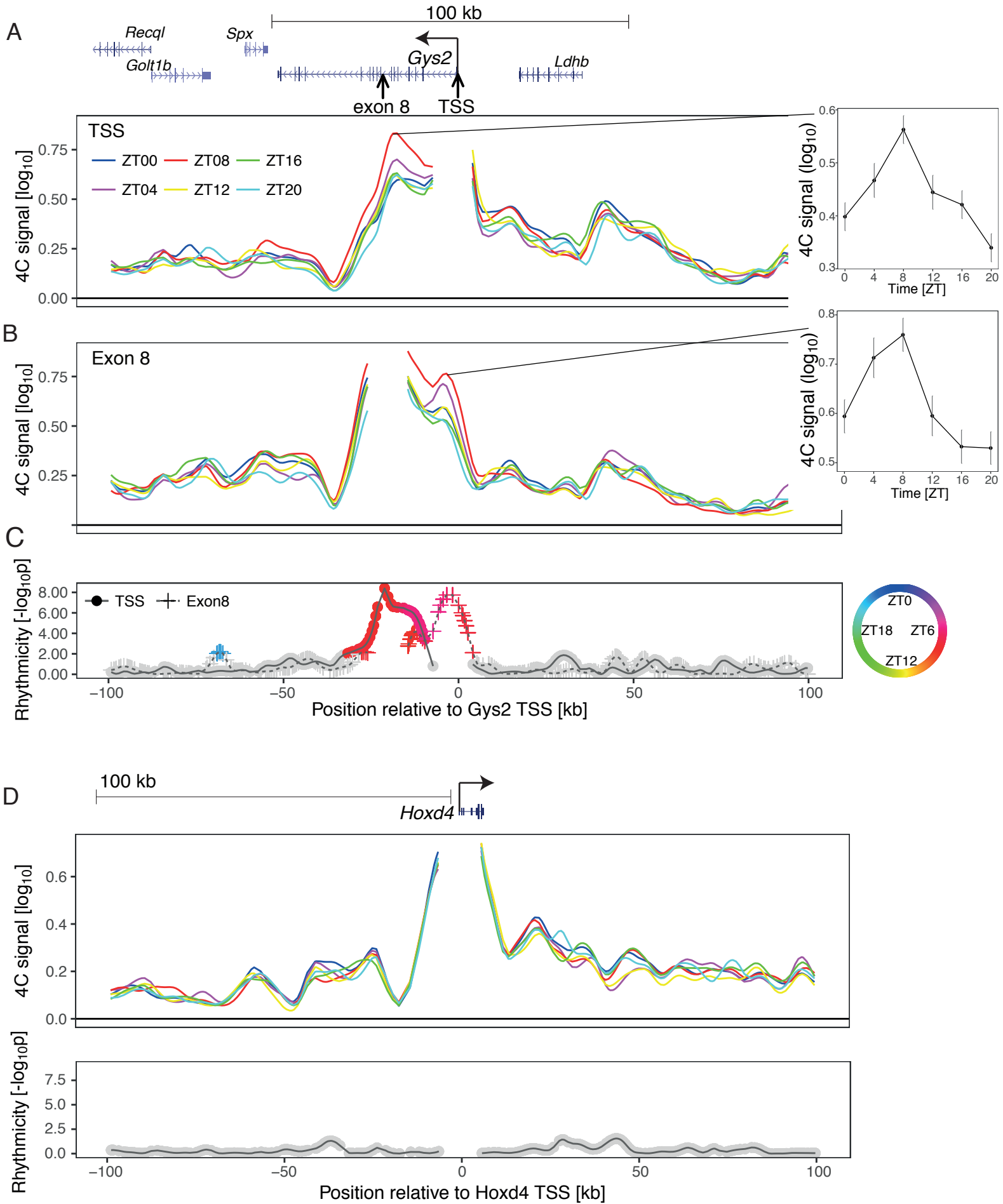

Supplement: Supplemental Material [file supp_gad.312397.118_Supplemental_Fig_S7_revised.pdf]

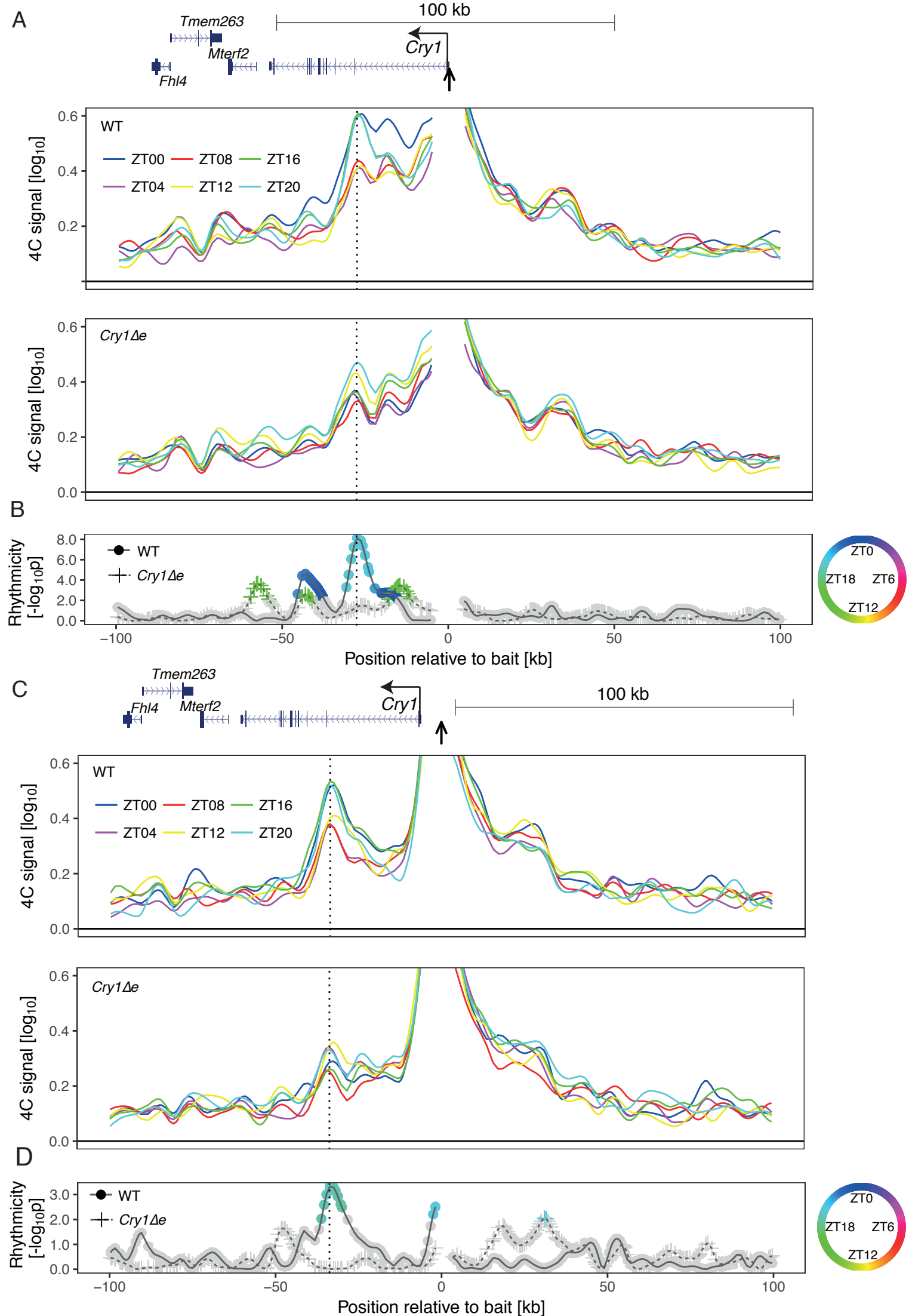

Supplement: Supplemental Material [file supp_gad.312397.118_Supplemental_Fig_S8_revised.pdf]

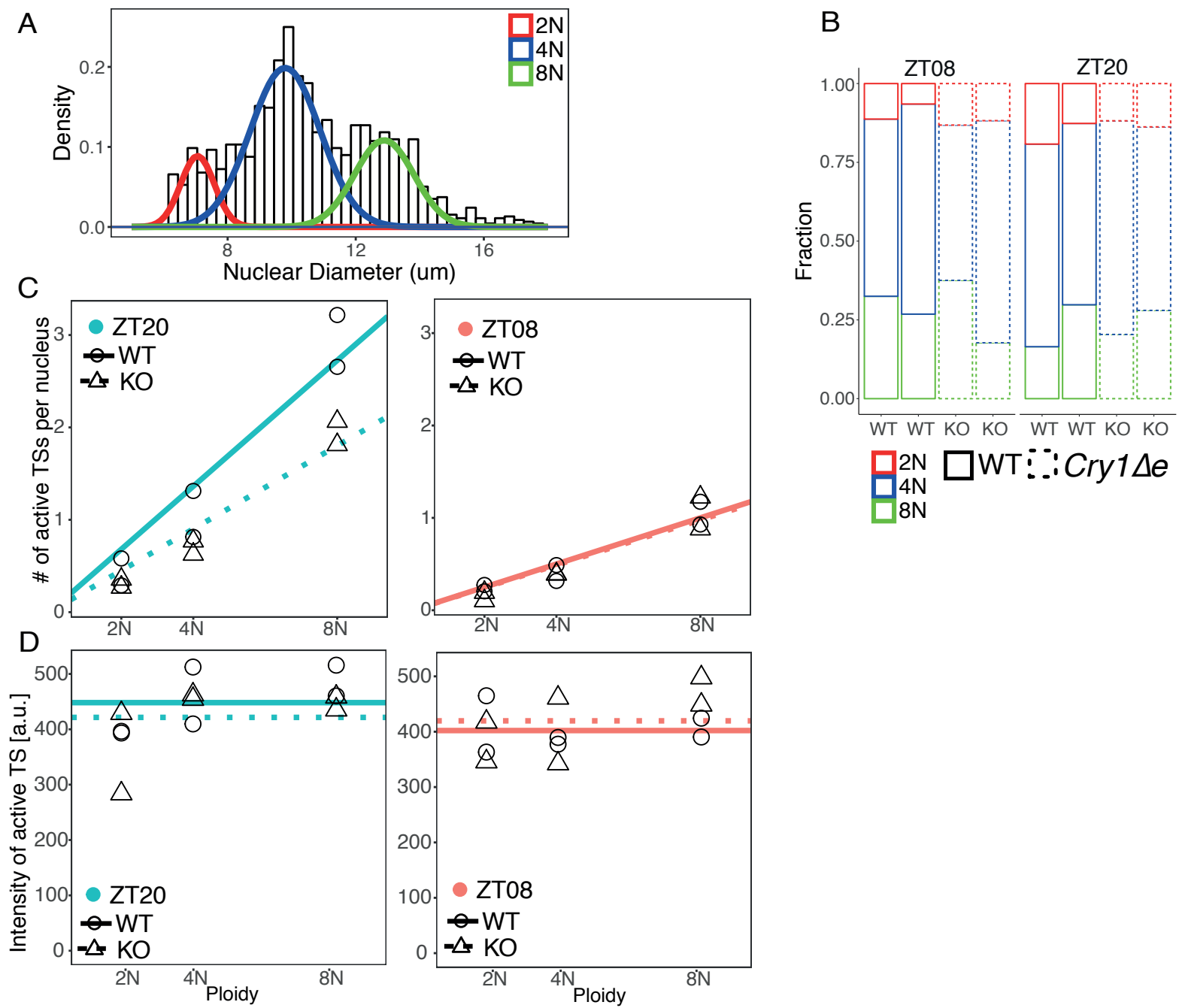

Supplement: Supplemental Material [file supp_gad.312397.118_Supplemental_Fig_S9_revised.pdf]
