# Supplementary material for: Clock-dependent chromatin topology modulates circadian transcription and behavior
Source: Genes Dev. 2018 Mar 1;32(5-6):347–58. doi: 10.1101/gad.312397.118 (PMC5900709; doi:10.1101/gad.312397.118)
Supplement: Supplemental Material [file supp_gad.312397.118_Supplemental_Captions.docx]

**Supplemental Figure S1: 4C-seq signal on *cis* and *trans* on baits targeting *Cry1* and *Gys2.*** (**A**) Temporal *Cry1* and *Gys2* mRNA accumulations in mouse liver (Atger et al., 2015) (reads per kilobase per million, RPKM); error bars: standard deviation (SD) of two animals; red and blue dashed lines: 4C-seq time points. (**B,C**) 4C-seq counts density vs. genomic position from *Cry1* TSS (B) and *Gys2* TSS (C) for each biological replicate at ZT08 (red) and ZT20 (blue) in the WT mouse liver. Dashed lines: power-law fit. α are decay exponents. Insets: cumulative counts on the *cis* chromosome from the bait position to chromosome end. Black vertical line shows 1 Mb from bait. (**D, E**) *Cry1* TSS (D) and *Gys2* TSS (E) 4C-seq counts density on *trans* chromosomes.

**Supplemental Figure S2: Validation of rhythmic chromatin interactions at *Cry1* and *Gys2* by targeting their respective enhancers**. (**A,B**) 4C-seq signals from the *Cry1* intron1 bait (A) and *Gys2* exon8 bait (B) in mouse liver at ZT08 (red) and ZT20 (blue) and the corresponding Z-score (ZT20-ZT08) and signed –log_10_(p) in a genomic window of 2 Mb. Largest differential signal occurs at the promoter region of the respective genes. Vertical arrows show location of bait relative to TSS of the respective gene.

**Supplemental Figure S3: 4C-seq in kidney show rhythmic chromatin interactions at *Cry1* TSS but not at *Gys2* TSS*.***

(**A**) *Cry1* mRNA profile in WT mouse liver (black) (Atger et al., 2015) and kidney (red); error bars: SD of two animals. (**B**) 4C-seq signal from *Cry1* TSS bait in WT kidney at ZT08 (red) and ZT20 (blue) in a genomic window of 200 kb. Vertical lines for *Cry1* show rhythmic contacts in kidney and liver. Z-score (ZT20 vs 08) and signed –log_10_(p) for rhythmic contacts in liver (black) and kidney (red). (**C**) Same as (B) using a 2 MB window. (**D**) same as (A) for *Gys2* mRNA. (**E**) same as (B) for *Gys2* TSS. Vertical lines for *Gys2* show contacts that are static in kidney but rhythmic in liver. (**F**) same as (C) for *Gys2* TSS*.*

**Supplemental Figure S4: Dynamics of chromatin topology depends on BMAL1.** (**A,B**) *Cry1* (A) and *Gys2* (B) mRNA profile in WT and *Bmal1* KO mouse liver along the diurnal cycle from (Atger et al., 2015); error bars: SD of two animals. (**C,D**) 4C-seq signals from *Cry1* TSS (C) and *Gys2* TSS (D) bait in WT and *Bmal1* KO livers at ZT08 and ZT20. Vertical lines show BMAL1-dependent rhythmic contacts. (**E**) 4C-seq signals from negative control region *Hoxd4* in WT and *Bmal1* KO livers show static chromatin topology. Bottom: Z-score (ZT20 vs 08) and signed –log_10_(p) for WT and *Bmal1* KO for *Hoxd4*.

**Supplemental Figure S5: Deleting *Cry1* intronic enhancer *in vivo* shortens the free-running period.** (**A**) Genome browser view showing the CRISPR-Cas9 mediated deletion of the *Cry1* intronic enhancer in mouse (*Cry1*Δe). The evolutionary conserved 300bp deletion (red square) covers the DHS containing a RRE described in mouse fibroblasts (Ukai-Tadenuma et al., 2011) within the H3K27ac marked intronic region. Note that this sequence is as conserved as *Cry1* exonic sequences. (**B**) Agarose gel showing the deletion. (**C-D**) Actograms showing the spontaneous locomotor activity recorded for 14 days in 12 hours/12 hours light/dark (LD) cycles followed by 21 days in constant darkness (DD) in a representative WT (C) and a *Cry1*Δe animal (D).

**Supplemental Figure S6: Deleting *Cry1* intronic enhancer *in vivo* shortens phase advances clock and clock-controlled gene expression.**

(**A**) PCA analysis of temporal RNA-seq data in the liver (left panel) and the kidney (right panel) of *Cry1*Δe and WT littermates. Right: color code for ZT time. (**B**) *Cry1* mRNA profile (in Transcripts Per Million, TPM) in liver (black) and kidney (red) of *Cry1*Δe (dashed line) and WT (solid line) littermates. Error bars: SD of 3 animals. At ZT20, *Cry1* mRNA levels are significantly different between WT versus *Cry1*Δe for liver and kidney (respectively p=0.045 and p=0.0037, t-test). *Cry1* mRNA is phase advanced in *Cry1*Δe versus WT (p=0.011 and p=0.047 for liver and kidney, respectively, bootstrap test). **(C)** *Cry1* intronic reads around the clock for WT and *Cry1*Δe mice. The oscillations in WT versus *Cry1*Δe are significantly different (p=0.0063, F-test) **(D,E)** Temporal expression of CRY1 protein in the liver cytoplasm of WT and *Cry1*Δe littermates. Western blot (D) and quantification of CRY1 relative abundance normalized by the Naptho blue black coloration of the membranes (E). CRY1 shows a decreased protein abundance in *Cry1*Δe (p=0.02, F-test). (**F**) Differential phase between *Cry1*Δe versus WT in liver and kidney. Genes selected for core-clock and clock-controlled transcripts (p<0.01, harmonic regression in both liver and kidney) based on Gene Ontology (GO) annotation indicated in Supplemental Table S5.

**Supplemental Figure S7: 4C-seq around the clock at *Gys2* TSS and at *Gys2* exon8 demonstrates rhythmic chromatin topology.**

(**A-B**) 4C-seq signals (LWMR summarizing n=3 animals per condition) from *Gys2* TSS (A) and exon8 (B) baits across time. Right: 4C signal over time at oscillatory chromatin contacts. (**C**) Statistical significance of rhythmic amplitudes of rhythmic contacts (p < 10^-7^ for TSS and exon8, chi-squared test). Fragments with p<0.01 are colored by their time of peak contact frequency according to color legend (right). (**D**) 4C-seq around the clock on negative control region, *Hoxd4,* in WT animals shows static chromatin topology.

**Supplemental Figure S8: Deleting the *Cry1* intronic enhancer disrupts rhythmic chromatin topology.** (**A**) 4C-seq signals (LWMR summarizing n=3 animals per condition) from *Cry1* TSS bait across time in WT (top panel) and *Cry1*Δe livers (middle panel). (**B**) Statistical significance of amplitudes of rhythmic contacts (­­chi-squared test, bottom panel) for *Cry1* TSS and *Cry1*Δe baits. Fragments with p<0.01 are colored by their time of peak contact frequency according to color legend (right). (**C**) *Idem* as (A) targeting the RRE bait -7 kb upstream *Cry1* TSS (*Cry1* upstream) (**D**) *Idem* as (B) for *Cry1* upstream. Vertical lines show rhythmic promoter-enhancer interactions that are disrupted in *Cry1*Δe liver. Vertical arrows show location of the bait relative to the *Cry1* TSS.

**Supplemental Figure S9: smRNA-FISH against *Cry1* pre-mRNA in WT versus *Cry1*Δe livers.** (**A**) Size distribution of nuclei for a representative animal. Colored curves show fitted Gaussian mixture model corresponding to populations with 2N, 4N, 8N ploidy. (**B**) Fraction of nuclei for each animal (n=2 per condition) assigned to different ploidy. (**C**) Number of active transcription sites (TSs) averaged per animal increases with ploidy. At ZT20, *Cry1*Δe animals show reduced number of TSs compared to WT: lines show mixed effect linear model with genotype-dependent slopes (p(H_0_:equal slopes)=0.00014, F-test); at ZT08, the slopes are not different (p=0.84). (**D**) Active TS intensity averaged per animal shows comparable intensity across ploidy and conditions: lines show mixed effect model with genotype-dependent intercepts, intercept comparisons at both ZT08 (p(H_0_:equal intercept)=0.53, F-test) and ZT20 (p=0.41) are not significant.

**Supplemental Table S1: Distribution of 4C-seq counts on the genome.** Sheet 1: Distribution of 4C-seq raw counts for each bait and each biological replicate in the genome. Exponent of the power-law fit is indicated. Sheet 2: $\sigma_{min}$ used to regularize the residual variance for each bait.

**Supplemental Table S2: Sequences of CRISPR-Cas9 RNA guides.** Sequences of CRISPR-Cas9 RNA guides targeting upstream and downstream the *Cry1* intronic enhancer element. The sequence of the PCR primers used to screen the deletion is indicated.

**Supplemental Table S3: 4C-seq primers.** Sequence of the PCR primers used for the inverse PCR step during 4C-seq libraries preparation.

**Supplemental Table S4: Motif counts.** List of motif counts for TF motifs from SwissRegulon. TFBS site count probabilities calculated using Motevo. Genomic coordinates from mm9 annotations.

**Supplemental Table S5: GO term annotation.** List of GO term annotation used to analyze temporal RNA-seq data in the liver and kidney of *Cry1*Δe and WT littermates.
